# Glycemic control among patients in China with type 2 diabetes mellitus receiving oral drugs or injectables

**DOI:** 10.1186/1471-2458-13-602

**Published:** 2013-06-21

**Authors:** Li-Nong Ji, Ju-Ming Lu, Xiao-Hui Guo, Wen-Ying Yang, Jian-Ping Weng, Wei-Ping Jia, Da-Jin Zou, Zhi-Guang Zhou, De-Min Yu, Jie Liu, Zhong-Yan Shan, Yu-Zhi Yang, Ren-Ming Hu, Da-Long Zhu, Li-Yong Yang, Li Chen, Zhi-Gang Zhao, Qi-Fu Li, Hao-Ming Tian, Qiu-He Ji, Jing Liu, Jia-Pu Ge, Li-Xin Shi, Yan-Cheng Xu

**Affiliations:** 1 Department of Endocrinology and Metabolism, Peking University People’s Hospital, No. 11 Xizhimen Nandajie, Beijing 100044, China; 2 Department of Endocrinology, Chinese PLA General Hospital, Beijing, China; 3 Department of Endocrinology, Peking University First Hospital, Beijing, China; 4 Department of Endocrinology, China-Japan Friendship Hospital, Beijing, China; 5 Department of Endocrinology, Third Affiliated Hospital of Sun Yat-Sen University, Guangzhou, China; 6 Department of Endocrinology, Shanghai Sixth People’s Hospital, Shanghai, China; 7 Department of Endocrinology, Changhai Hospital, Second Military Medical University, Shanghai, China; 8 Department of Endocrinology, Second Xiangya Hospital of Central South University, Changsha, China; 9 Department of Endocrinology, Tianjin Medical University Metabolic Disease Hospital, Tianjin, China; 10 Department of Endocrinology, Shanxi Provincial People’s Hospital, Taiyuan, China; 11 Department of Endocrinology, First Hospital of China Medical University, Shenyang, China; 12 Department of Endocrinology, Heilongjiang Province Hospital, Harbin, China; 13 Department of Endocrinology, Huashan Hospital, Fudan University, Shanghai, China; 14 Department of Endocrinology, Nanjing Drum Tower Hospital, Affiliated Hospital of Nanjing University Medical School, Nanjing, China; 15 Department of Endocrinology, First Affiliated Hospital of Fujian Medical University, Fuzhou, China; 16 Department of Endocrinology, Qilu Hospital of Shandong University, Jinan, China; 17 Department of Endocrinology, Henan Provincial People’s Hospital, Zhengzhou, China; 18 Department of Endocrinology, First Affiliated Hospital of Chongqing Medical University, Chongqing, China; 19 Department of Endocrinology, West China Hospital, Sichuan University, Chengdu, China; 20 Department of Endocrinology, Xijing Hospital, Fourth Military Medical University, Xi’an, China; 21 Department of Endocrinology, Gansu Provincial Hospital, Lanzhou, China; 22 Department of Endocrinology, Xinjiang Uigur Autonomous Region People’s Hospital, Urumqi, China; 23 Department of Endocrinology, Affiliated Hospital of Guiyang Medical College, Guiyang, China; 24 Department of Endocrinology, Zhongnan Hospital of Wuhan University, Wuhan, China

**Keywords:** China, GLP-1 receptor agonists, HbA1c, Insulin, Oral antidiabetes drugs (OADs), Type 2 diabetes mellitus

## Abstract

**Background:**

The prevalence of type 2 diabetes mellitus (T2DM) is increasing rapidly among Chinese adults, and limited data are available on T2DM management and the status of glycemic control in China. We assessed the efficacy of oral antidiabetes drugs (OADs), glucagon-like peptide-1 (GLP-1) receptor agonists, and insulin for treatment of T2DM across multiple regions in China.

**Methods:**

This was a multicenter, cross-sectional survey of outpatients conducted in 606 hospitals across China. Data from all the patients were collected between April and June, 2011.

**Results:**

A total of 238,639 patients were included in the survey. Eligible patients were treated with either OADs alone (n=157,212 [65.88%]), OADs plus insulin (n=80,973 [33.93%]), or OADs plus GLP-1 receptor agonists (n=454 [0.19%]). The OAD monotherapy, OAD + insulin, and OAD + GLP-1 receptor agonist groups had mean glycosylated hemoglobin (HbA1c) levels (±SD) of 7.67% (±1.58%), 8.21% (±1.91%), and 7.80% (±1.76%), respectively. Among those three groups, 34.63%, 26.21%, and 36.12% met the goal of HbA1c <7.0%, respectively. Mean HbA1c and achievement of A1c <7.0% was related to the duration of T2DM.

**Conclusions:**

Less than one third of the patients had achieved the goal of HbA1c <7.0%. Glycemic control decreased and insulin use increased with the duration of diabetes.

## Background

According to a 1994 study, the incidence of type 1 diabetes mellitus (T1DM), also known as insulin-dependent diabetes or childhood diabetes, in Shanghai, China is 0.72 per 100,000 residents, making it among the lowest in the world [1]. At this incidence rate, the 13,000 T1DM cases diagnosed annually in the United States would drop to <500 new cases per year [1]. The prevalence of both prediabetes and type 2 diabetes mellitus (T2DM) in Chinese adults, however, is much higher, with a recent national survey showing a 2008 prevalence rate of 9.7% for subjects ≥20 years old [2,3]. The prevalence of prediabetes, defined by either impaired fasting glucose or impaired glucose tolerance, is estimated to be 15.5% in Chinese adults, which would make the total adult population with prediabetes in China about 148.2 million people (76.1 million men and 72.1 million women) [2]. T2DM is estimated to affect 9.7% of the Chinese population (10.6% of men and 8.8% of women), which would make 92.4 million adults in China who have diabetes (50.2 million men and 42.2 million women) [2].

Several large, well-known studies, including the United Kingdom Prospective Diabetes Study (UKPDS), have demonstrated the importance of glycemic control among patients with T2DM [4-6]. Such research has shown there is a strong correlation between mean glycated hemoglobin (HbA1c) levels over time and the development and progression of diabetic complications. Both the American Diabetes Association and the Chinese Diabetes Society advocate a HbA1c goal of <7.0% for individuals with T2DM [7,8], although research has demonstrated that glycemic control is difficult to achieve in China and other Asian countries [2,3,9,10]. It has been previously shown that approximately one half of the outpatients with T2DM who are treated in the metropolitan medical centers in China have inadequate glycemic control when they are treated with oral antidiabetic drugs (OADs) alone [11]. Currently there are several types of treatment for T2DM, including OADs such as metformin, sulfonylureas, thiazolidinediones, alpha-glucosidase inhibitors, meglitinides, and dipeptidyl peptidase-4 (DPP-4) inhibitors; glucagon-like peptide-1 (GLP-1) receptor agonists; and insulin; and they can be used as monotherapy or in combinations to arrive at the best individualized treatment plan to achieve treatment goals [8,12,13]. Intensification of treatment is often required over time as the disease progresses, with the addition of multiple OADs and/or insulin.

A study of 7549 Chinese patients with T2DM in the Hong Kong Diabetes Registry found that among those treated with OADs and/or insulin, only 39.7% attained the glycemic target of HbA1c <7% and that both long disease duration and complexity of treatment regimens were associated with suboptimal glycemic control [14]. The International Diabetes Mellitus Practice Study (IDMPS) was a 5-year survey that documented changes in diabetes treatment practice in developing regions and employed logistic regression analysis to identify factors for achieving HbA1c <7% in 11,799 patients, 1898 with T1DM and 9901 with T2DM; patients were recruited from 17 countries in Eastern Europe, Asia, Latin America, and Africa [15]. That study revealed that only 20%-30% of patients achieved the goal of HbA1c <7%. An additional publication of results from IDMPS reported that education increased the use of insulin and improved self-care performance of patients with T2DM, and that it resulted in lower rates of chronic complications while significantly increasing the percentage of individuals patients who achieved HbA1c <7% [16].

Although the population with T2DM in China is close to 100 million patients, limited data are available on the management of those individuals and the status of glycemic control within that nation. The objective of this noninterventional study was to evaluate the characteristics, glycemic status, and treatment strategies used in patients with T2DM across China treated with oral agents alone or in conjunction with or GLP-1 receptor agonists.

## Methods

### Study design

This was a multicenter, cross-sectional survey of outpatients with T2DM conducted in 606 hospitals across China, representing every region of the mainland other than Tibet. Each of the patients provided written informed consent. All of the data were collected between April and June, 2011. Prior to initiation of this study, the survey used (see Additional file 1) was approved by the Ethics Committee of Chinese PLA General Hospital, and was reviewed and accepted by all the participating hospitals.

Each day during the study period, the first 7 patients who entered a participating facility and met the eligibility criteria were invited to participate. The study investigators conducted the surveys by communicating directly with the patients, and interviews were performed by study investigators to complete the survey. All laboratory evaluations were performed in the local hospitals where the interviews were conducted.

The survey captured general information about each patient, including gender, height, weight, blood pressure, and lipid profile. Laboratory data on HbA1c, fasting plasma glucose (FPG), and postprandial plasma glucose (PPG) levels were collected. Specific information about the treatments used for the management of their T2DM were identified, including the use of OADs (including DPP-4 inhibitors), GLP-1 receptor agonists, and different types of insulin, as well as combinations of OADs and insulin and the combination of OADs and a GLP-1 receptor agonist.

Patients were also required to report if they were diagnosed with any concomitant diseases or diabetes complications, including hypertension, coronary heart disease, dyslipidemia, cerebrovascular disease, diabetic retinopathy, diabetic neuropathy, diabetic nephropathy, diabetes-related foot ulcers, and others (see Additional file 1).

### Inclusion criteria

Outpatients with T2DM being treated with OADs alone, OADs combined with insulin, or OADs combined with GLP-1 receptor agonists were eligible to participate in this study if they were at least 18 years of age, had at least one previous outpatient medical record pertaining to diabetes, and had resided within a local area for at least 6 consecutive months prior to initiation of the study.

### Exclusion criteria

Individuals could not participate in this study if they had secondary diabetes [17] or were only receiving treatment with insulin. Patients were excluded if they were not receiving OAD monotherapy, OADs in combination with insulin, or OADs in combination with GLP-1 receptor agonists. Other individuals who were excluded were patients with T1DM, inpatients, those receiving therapeutic lifestyle changes or Chinese herbal medicine only, those who were pregnant or breast-feeding, and those who were either mentally incapable or for other reasons unable to adequately understand or participate in the study.

### Statistical analyses

Statistical analyses were conducted using SAS version 9.1.3 (SAS Institute, Cary, NC, USA). Descriptive statistics were used to characterize the data in the study, including calculations of means and standard deviations. Comparisons were statistically analyzed using *t*-tests and chi-squared tests, and a logistic regression analysis of patient characteristics and treatment regimens was conducted.

## Results

A total of 238,656 subjects with T2DM were surveyed and 238,639 were included in the final analyses. Seventeen subjects who completed the survey but had received monotherapy with GLP-1 receptor agonists were excluded. Demographic information and baseline characteristics of the study population are shown in Table 1. Table 2 presents the clinical characteristics of the patients, including their treatment regimens and HbA1c levels. Mean HbA1c did not vary by BMI at baseline; however, a slightly higher percentage of patients with BMI <24 kg/m^2^ achieved HbA1c <7.0% than patients with BMI ≥24 kg/m^2^. Mean HbA1c was significantly lower and HbA1c goal attainment was significantly higher among patients who performed self-monitored blood glucose (SMBG) compared to those who did not. Compared to those without diabetes complications or concomitant disease, individuals who had them had higher HbA1c levels (and a lower rate of HbA1c goal achievement (Table 2).

**Table 1:** Baseline characteristics

**Characteristic**	**Male**	**Female**	**All**
	**Mean±SD**		
Age, yrs	58.39±12.01	59.09±11.32	58.72±11.69
BMI, kg/m^2^	24.47±2.92	24.38±3.41	24.43±3.16
Duration of diabetes, yr	5.43±5.16	5.71±5.40	5.57±5.28
HbA1c, %	7.90±1.73	7.80±1.71	7.85±1.72
FPG, mmol/L	8.12±2.40	8.02±2.39	8.07±2.39
2hPPG, mmol/L	11.26±3.64	11.12±3.63	11.19±3.64
SBP, mmHg	132.35±14.79	131.39±15.50	131.89±15.14
DBP, mmHg	81.86±11.00	80.10±11.05	81.02±11.06
Triglycerides, mmol/L	2.22±1.65	2.10±1.46	2.16±1.56
Total cholesterol, mmol/L	4.71±1.48	4.75±1.48	4.73±1.48
LDL, mmol/L	2.92±1.16	2.92±1.16	2.92±1.16
**Reason for insulin initiation, n (%)**			
OAD ineffective	33341 (77.18)	29525 (78.17)	62866 (77.64)
Complication	5531 (12.80)	4862 (12.87)	10393 (12.84)
Patient requests	3041 (7.04)	2382 (6.31)	5423 (6.70)
Other reason	1288 (2.98)	1003 (2.65)	2291 (2.82)
**Complications and comorbidities, n (%)**			
Hypertension	41400 (33.25)	38194 (33.46)	79594 (33.35)
Coronary heart disease	12918 (10.38)	13125 (11.50)	26043 (10.91)
Dyslipidemia	25222 (20.26)	21712 (19.02)	46934 (19.67)
Cerebrovascular disease	6552 (5.26)	5526 (4.84)	12078 (5.06)
Diabetic retinopathy	9799 (7.87)	10046 (8.80)	19845 (8.32)
Diabetic neuropathy	13211 (10.61)	12930 (11.33)	26141 (10.95)
Diabetic nephropathy	7001 (5.62)	5702 (5.00)	12703 (5.32)
Diabetic foot	1380 (1.11)	1087 (0.95)	2467 (1.03)
Others	2438 (1.96)	2469 (2.16)	4907 (2.06)

2hPPG, 2-hr postprandial plasma glucose; BMI, body mass index; DBP, diastolic blood pressure; FPG, fasting plasma glucose; HbA1c, glycated hemoglobin; LDL, low-density lipoprotein; SBP, systolic blood pressure; SD, standard deviation.

**Table 2:** Clinical characteristics

**Characteristic**		**Diabetes duration (yrs)**	**Age (yrs)**	**HbA1c (%)**	**Patients with HbA1c <7.0%**
	**n (%)**	**Mean±SD**			**n (%)**
No. of patients	238,639 (100)	5.57±5.28	58.72±11.69	7.85±1.72	75,829 (31.78)
BMI					
<24 kg/m^2^	110,283 (46.21)	5.44±5.26	58.43±11.73	7.85±1.79	36,507 (33.10)
≥24 kg/m^2^	128,356 (53.79)	5.67±5.29	58.98±11.66	7.86±1.67	39,322 (30.64)
P value		<0.0001	<0.0001	0.2848	<0.0001
Treatment regimen					
OAD only	157,212 (65.88)	4.76±4.65	58.16±11.52	7.67±1.58	54,438 (34.63)
1 OAD	58,028 (24.32)	4.38±4.95	57.45±12.08	7.65±1.69	21,834 (37.63)
2 OADs	80,113 (33.57)	4.74±4.34	58.35±11.21	7.66±1.53	26,942 (33.63)
≥3 OADs	19,071 (7.99)	6.01±4.71	59.56±10.89	7.75±1.49	5662 (29.69)
P value		<0.0001	<0.0001	<0.0001	<0.0001
OAD + insulin	80,973 (33.93)	7.12±6.04	59.85±11.92	8.21±1.91	21,227 (26.21)
Prandial	6243 (2.62)	6.30±5.91	58.83±12.49	8.14±2.06	1745 (27.95)
Basal	13,816 (5.79)	6.58±5.54	59.13±11.97	7.99±1.81	4251 (30.77)
Premixes	53,122 (22.26)	7.20±6.02	60.24±11.66	8.18±1.86	13,787 (25.95)
Basal-bolus	6411 (2.69)	8.12±6.87	58.90±13.02	8.89±2.25	1143 (17.83)
Others	1381 (0.58)	8.70±6.75	60.70±12.57	8.45±2.00	301 (21.80)
P value		<0.0001	<0.0001	<0.0001	<0.0001
OAD + GLP-1 RA	454 (0.19)	5.39±4.46	53.12±12.90	7.80±1.76	164 (36.12)
P value^a^		<0.0001	<0.0001	<0.0001	<0.0001
SMBG					
Yes	90,557 (37.95)	5.86±5.25	58.96±11.46	7.68±1.62	31,979 (35.31)
No	148,082 (62.05)	5.39±5.29	58.58±11.83	7.96±1.77	43,850 (29.61)
P value		<0.0001	<0.0001	<0.0001	<0.0001
Concomitant disease/complications					
Yes	124,182 (52.04)	6.82±5.70	61.13±11.36	7.95±1.77	36,947 (29.75)
No	81,405 (34.11)	4.09±4.39	55.30±11.38	7.71±1.69	29,254 (35.94)
Don’t know	33,052 (13.85)	4.47±4.39	58.09±11.48	7.82±1.58	9,628 (29.13)
P value		<0.0001	<0.0001	<0.0001	<0.0001

^a^Comparison among different treatment regimens, ie, OAD only, OAD + insulin, OAD + GLP-1 RA.

P values were calculated using chi-square analysis and ANOVA as appropriate.

BMI, body mass index; GLP-1 RA, glucagon-like peptide-1 receptor agonist; HbA1c, glycated hemoglobin; OAD, oral antidiabetes drug; SD, standard deviation; SMBG, self-monitored blood glucose.

Mean HbA1c and HbA1c goal attainment varied among treatment groups. Mean HbA1c was lower and the proportion of patients who achieved HbA1c <7.0% was higher among patients receiving OADs only and patients receiving OADs plus GLP-1 receptor agonists compared to patients receiving insulin plus OADs (Table 2). In fact, mean HbA1c increased and HbA1c goal achievement decreased with increasing numbers of OADs used. Among patients receiving OADs and insulin, mean HbA1c was lowest among patients receiving basal insulin alone and highest among patients receiving basal-bolus therapy. HbA1c goal achievement followed the same trend. However, diabetes duration was shorter among patients using OADs only (4.76 years) and longer among patients using OADs and insulin (7.12 years; p < 0.0001). Diabetes duration increased with the number of OADs used and increased as insulin therapy intensified (Table 2).

**Figure 1:**
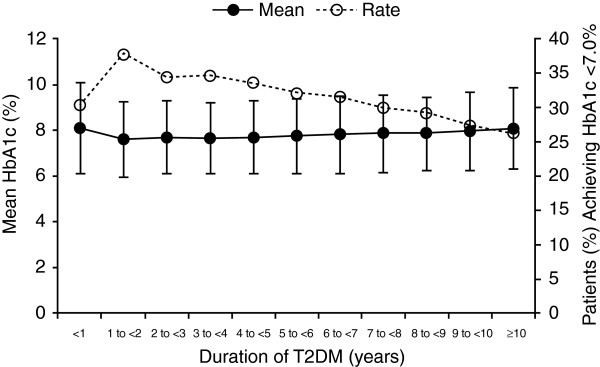
**Mean HbA1c and mean percentage of patients achieving HbA1c <7.0% by duration of type 2 diabetes.** HbA1c: glycated hemoglobin, T2DM: type 2 diabetes mellitus.

The association between diabetes duration, diabetes complications and concomitant diseases, treatment, and glycemic control is explored in more detail in Table 3. Regardless of treatment used, mean HbA1c generally increased with the duration of diabetes, as also shown in Figure 1. Similarly, HbA1c goal achievement generally decreased with the duration of diabetes (Table 3). The exception to both these trends occurred among individuals with a diabetes duration of <1 year, and the prevalence of concomitant diseases and complications also increased with diabetes duration.

**Table 3:** Patient characteristics by duration of diabetes

**Diabetes duration (yrs)**	**n (%)**^**a**^	**Mean±SD HbA1c (%)**	**Concomitant disease/ complication (%)**^**b**^	**Achieved HbA1c <7.0% (%)**^**b**^	**OAD (%)**^**b**^	**Insulin + OAD (%)**^**b**^	**GLP-1 RA + OAD (%)**^**b**^
<1	36,337 (15.22)	8.12±1.99	12,663 (34.85)	11,007 (30.29)	26,372 (72.58)	9909 (27.27)	56 (0.15)
≥1-5	102,550 (42.97)	7.66±1.61	45,533 (44.40)	36,331 (35.43)	75,459 (73.58)	26,896 (26.23)	195 (0.19)
≥5-10	55,744 (23.36)	7.84±1.66	34,374 (61.67)	16,915 (30.34)	34,446 (61.79)	21,159 (37.96)	139 (0.25)
≥10	44,003 (18.44)	8.09±1.76	31,607 (71.83)	11,575 (26.31)	20,933 (47.57)	23,006 (52.28)	64 (0.15)
P value^**c**^		<0.0001	<0.0001	<0.0001	<0.0001	NS	NS

^a^Percentage of patients among the full population; ^b^Percentage of patients among the patients with particular diabetes duration; ^**c**^ANOVA and chi-square test were used as appropriate.

GLP-1 RA, glucagon-like peptide-1 receptor agonist; HbA1c, glycated hemoglobin; NS = not significant; OAD, oral antidiabetes drug; SD, standard deviation.

Trends in treatment with diabetes duration were also observed. The use of oral agents alone decreased with diabetes duration (from 72.58% in individuals with a duration of <1 year to 47.57% in individuals with a diabetes duration of ≥10 years). Corresponding increases in OAD plus insulin use were also observed. Among individuals with a diabetes duration of <1 year, 27.27% used OADs in combination with insulin, while 52.28% of individuals with a diabetes duration of ≥10 years used a combination of OADs and insulin (Table 3).

The survey also captured reasons why insulin was added to the OAD regimens (data not shown). OADs were deemed ineffective (62,866/80,973; 77.64%), there were complications (10,393/80,973; 12.84%), and other reasons, not specified. Among the small numbers of patients who used GLP-1 receptor agonists with OADs, frequency of use did not appear to vary by diabetes duration.

Table 4 presents data on the type of insulin used among individuals reporting combination OAD and insulin use by the duration of diabetes. Overall, approximately two thirds (52,643/80,308, 65.6%) of OAD plus insulin users used insulin premixes. The proportion of insulin premix use was relatively consistent regardless of the duration of diabetes, ranging from 62.91% to 67.50% with no discernible pattern across the duration of the disease. No overt trends in prandial or basal insulin use by diabetes duration were observed, although there was a small increase in the use of basal-bolus therapy with increasing diabetes duration. A logistic regression analysis revealed the several characteristics and treatment regimens that were associated with patients attaining HbA1c <7.0% (Table 5).

**Table 4:** Insulin use in OAD plus insulin group, by insulin treatment duration

**Insulin treatment duration (yrs)**	**n (%)**^**a**^	**Prandial, n (%)**^**b**^	**Basal, n (%)**^**b**^	**Basal-bolus, n (%)**^**b**^	**Premixes, n (%)**^**b**^	**Others, n (%)**^**b**^
<1	21,145	1987	3376	1623	13,825	334
	(26.33)	(9.40)	(15.97)	(7.68)	(65.37)	(1.58)
≥1-5	27,617	2171	4992	1711	18,334	409
	(34.39)	(7.86)	(18.08)	(6.20)	(66.38)	(1.48)
≥5-10	18,504	1198	3148	1561	12,280	317
	(23.04)	(6.47)	(17.01)	(8.44)	(66.37)	(1.71)
≥10	13,042 (16.24)	834 (6.39)	2,223 (17.04)	1471 (11.28)	8204 (62.91)	310 (2.38)
Total^c^	80,308	6190	13,739	6366	52,643	1370
P value^d^		<0.0001	NS	NS	NS	NS

^a^Percentage of patients among the full population; ^b^Percentage of patients among the patients with particular insulin treatment duration; ^c^Data on diabetes duration were missing for 665 patients using insulin therapy; ^d^Chi-square test was used to compare the percentage of patients in different insulin regimens by insulin treatment duration.

NS = not significant.

**Table 5:** Logistic regression analysis for the patients achieving HbA1c <7.0%

**Characteristics**	**Wald X**^**2**^	**P value**	**Odds ratio**	**95% CI**
Age	163.1497	<0.0001	1.005	1.004-1.006
Sex male	187.2576	<0.0001	0.885	0.870-0.901
Duration of diabetes	468.9675	<0.0001	0.979	0.977-0.981
BMI	101.4330	<0.0001	0.986	0.983-0.988
No complications	321.8294	<0.0001	1.203	1.179-1.228
SMBG	1251.9703	<0.0001	1.387	1.362-1.412
Treatment regimen (vs OAD+insulin)				
OAD+GLP1	22.6761	<0.0001	1.600	1.319-1.941
1 OAD	1663.4200	<0.0001	1.649	1.610-1.690
2 OADs	804.4268	<0.0001	1.377	1.347-1.408
≥3 OADs	88.2917	<0.0001	1.183	1.142-1.225

Results interpretation: Patients who are elderly, female, with shorter duration of diabetes, less BMI, without complications, performed SMBG recently, treated with OAD only or OAD+GLP1 were more likely to attain the HbA1c targets.

BMI, body mass index; CI, confidence interval; GLP-1 RA, glucagon-like peptide-1 receptor agonist; OAD, oral antidiabetes drug; SMBG, self-monitored blood glucose.

## Discussion

Results from this large-scale survey of almost 240,000 patients throughout China demonstrated that, overall, patients with T2DM do not meet the treatment guideline set by the American Diabetes Association (ADA) and the Chinese Medical Society of an HbA1c <7%. More specifically, less than one third of individuals with type 2 diabetes using OADs, either alone or in combination with insulin or GLP-1 receptor agonists, achieved glycemic control as defined by HbA1c <7.0%. These results are comparable to those obtained in an earlier study that reported 39.7% of patients treated with OADs and/or insulin achieved HbA1c <7% [14]. Because our objective was to characterize Chinese patients receiving OADs for T2DM in China, our analysis did not include patients treated with diet and lifestyle interventions alone or insulin without oral agents.

Glycemic control appeared to be greater among individuals treated with only OADs compared to those receiving more intensive therapy with OADs in combination with insulin. This, however, might be a function of duration of disease. Similarly, among patients using OADs in combination with insulin, glycemic control appeared to be greater among patients receiving prandial insulin alone, basal insulin alone, or insulin premixes compared with basal-bolus therapy, a more intensive intervention. This difference may represent an attempt to improve glycemic control in patients at higher HbA1c levels through the addition of more intensive insulin therapy.

It is also likely that the difference in glycemic control may result from differences in disease severity. This hypothesis is supported by several findings in our study. First, mean HbA1c generally increased and HbA1c goal achievement generally decreased with increasing diabetes duration. The exception in this trend occurred in patients with a diabetes duration of less than 1 year, possibly because the optimal treatment regimen had not yet been identified due to the recentness of the diagnosis. Both the association between glycemic control and diabetes duration, and the differences in glycemic control between individuals using OADs and those using insulin, have been reported in other observational studies, including a recent study of Chinese individuals with type 2 diabetes in the Jiangsu province [18]. It also should be noted that differences in glycemic control among study groups were relatively small, suggesting that treatment was adjusted to accommodate the progressive hyperglycemia seen with advancing diabetes.

Because diabetes is a progressive disease, patients who have had a longer duration of diabetes are likely to have reduced beta-cell function and require more intensive therapy compared to patients with more recently diagnosed disease. In our study, patients using OADs plus insulin therapy had a longer history of diabetes than patients using OADs alone. This trend was apparent even among patients using only OADs: patients using 3 or more OADs had a longer duration of diabetes than patients using only one OAD.

The inverse association between disease severity and glycemic control is also supported by the prevalence of concomitant diseases and diabetes complications in the study. Patients using OADs plus insulin typically had a higher prevalence of complications and concomitant disease than patients using only OADs. Not surprisingly, the prevalence of complications and concomitant disease also increased with diabetes duration, a finding indicative of the association between duration of hyperglycemia and likelihood of adverse vascular effects.

Our study also demonstrated the positive impact of SMBG on glycemic control. Patients who performed SMBG had a lower mean HbA1c and a greater percentage of HbA1c goal achievement compared to patients who did not. Interestingly, patients who performed SMBG had a longer history of diabetes compared with those who did not. This may be due to the increased need for SMBG with the use of more intensive insulin regimens utilized by patients with more advanced disease. Nonetheless, these findings suggest that initiating SMBG may be one way to overcome the loss of diabetes control with diabetes progression despite the increased intensity of treatment.

This study has several limitations. First, this is a descriptive analysis of the results from patient interviews. No attempt was made to control for confounding factors, and statistical analyses of the data were limited. Results collected from patient interviews and self-reports are subject to bias; however, patient interviews are the most practical way of obtaining such information in China [18].

## Conclusions

This is one of the largest studies conducted among the Chinese population with T2DM, and the data obtained during this study reveal several clinically important findings. The majority of patients with type 2 diabetes receiving oral agents did not achieve the goal of HbA1c <7.0%, indicating that substantial improvements in treatment are still necessary. In fact, among all three patient groups, those receiving OADs, OADs in combination with GLP-1 receptor agonists, and OADs in combination with insulin, only 26%-38% achieved the goal of HbA1c <7.0%. Further research could reveal that adjusting or changing treatments earlier or more frequently may enable patients to achieve even better glycemic control.

## Abbreviations

2hPPG: 2-hr postprandial plasma glucose; ADA: American Diabetes Association; BMI: Body mass index; DBP: Diastolic blood pressure; DPP-4: Dipeptidyl peptidase-4; FPG: Fasting plasma glucose; GLP-1: Glucagon-like peptide-1; GLP-1 RA: GLP-1 receptor agonist; LDL: Low-density lipoprotein; OADs: Oral antidiabetes drugs; PPG: Postprandial plasma glucose; SBP: Systolic blood pressure; SD: Standard deviation; SMBG: Self-monitored blood glucose; T1DM: type 1 diabetes mellitus; T2DM: type 2 diabetes mellitus; UKPDS: UK Prospective Diabetes Study.

## Competing interests

The authors declare that they have no competing interests. Research funding to support this study was provided to the investigators’ institutions by Novo Nordisk (China) Pharmaceutical Co., Ltd.

## Authors’ contributions

All the authors have made a significant contribution to this manuscript, have read and approved the final manuscript, and have agreed to its submission*.* LNJ designed, conducted the study, and drafted the manuscript; JML and XHG conducted the study and participated in the design and interpretation of the results; WYY, JPW, WPJ, DJZ, ZGZ, DMY, JL, ZYS, YZY, RMH, DLZ, LYY, LC, ZGZ, QFL, HMT, QHJ, JL, JPG, LXS, and YCX conducted the study. All authors participated in the preparation of the manuscript and approved the final version for submission.

## Pre-publication history

The pre-publication history for this paper can be accessed here:

http://www.biomedcentral.com/1471-2458/13/602/prepub

## Supplementary Material

Additional file 1 HbA1c Surveillance Registration Form. Click here for file

## References

[B1] Fu H Shen SX Chen ZW Wang JJ Ye TT LaPorte RE Tajima N Shanghai, China, has the lowest confirmed incidence of childhood diabetes in the world Diabetes Care 1994 17 1206 1208 10.2337/diacare.17.10.1206 7821146

[B2] Yang W Lu J Weng J Jia W Ji L Xiao J Shan Z Liu J Tian H Ji Q Zhu D Ge J Lin L Chen L Guo X Zhao Z Li Q Zhou Z Shan G He J Prevalence of diabetes among men and women in China N Engl J Med 2010 362 1090 1101 10.1056/NEJMoa0908292 20335585

[B3] Zhao D Zhao F Li Y Zheng Z Projected and observed diabetes epidemics in China and beyond Curr Cardiol Rep 2012 14 106 111 10.1007/s11886-011-0227-9 22057734

[B4] UK Prospective Diabetes Study Group Intensive blood-glucose control with sulphonylureas or insulin compared with conventional treatment and risk of complications in patients with type 2 diabetes (UKPDS 33) Lancet 1998 352 837 853 9742976

[B5] Skyler JS Bergenstal R Bonow RO Buse J Deedwania P Gale EA Howard BV Kirkman MS Kosiborod M Reaven P Sherwin RS Intensive glycemic control and the prevention of cardiovascular events: implications of the ACCORD, ADVANCE, and VA diabetes trials. A position statement of the American Diabetes Association and a scientific statement of the American College of Cardiology Foundation and the American Heart Association Diabetes Care 2009 32 187 192 10.2337/dc08-9026 19092168 PMC2606812

[B6] UK Prospective Diabetes Study Group Effect of intensive blood-glucose control with metformin on complications in overweight patients with type 2 diabetes (UKPDS 34) Lancet 1998 352 854 865 9742977

[B7] Chinese Diabetes Society China guideline for type 2 diabetes Chin J Diabetes Mellitus 2010 2 suppl 2 6 56

[B8] American Diabetes Association Standards of medical care in diabetes–2013 Diabetes Care 2013 36 suppl 1 S11 S66 23264422 10.2337/dc13-S011 PMC3537269

[B9] So WY Raboca J Sobrepena L Yoon KH Deerochanawong C Ho LT Himathongkam T Tong P Lyubomirsky G Ko G Nan H Chan J Comprehensive risk assessments of diabetic patients from seven Asian countries: the Joint Asia Diabetes Evaluation (JADE) program J Diabetes 2011 3 109 118 10.1111/j.1753-0407.2011.00115.x 21599865

[B10] Editorial China's major health challenge: control of chronic diseases Lancet 2011 378 457 10.1016/S0140-6736(11)61232-4 21821170

[B11] Zhang SL Chen ZC Yan L Chen LH Cheng H Ji LN Determinants for inadequate glycaemic control in Chinese patients with mild-to-moderate type 2 diabetes on oral antidiabetic drugs alone Chinese Med J (Engl) 2011 124 2461 2468 21933588

[B12] Rodbard HW Jellinger PS Davidson JA Einhorn D Garber AJ Grunberger G Handelsman Y Horton ES Lebovitz H Levy P Moghissi ES Schwartz SS Statement by an American Association of Clinical Endocrinologists/American College of Endocrinology consensus panel on type 2 diabetes mellitus: an algorithm for glycemic control Endocr Pract 2009 15 540 559 10.4158/EP.15.6.540 19858063

[B13] Inzucchi SE Bergenstal RM Buse JB Diamant M Ferrannini E Nauck M Peters AL Tsapas A Wender R Matthews DR Management of hyperglycemia in type 2 diabetes: a patient-centered approach. Position statement of the American Diabetes Association (ADA) and the European Association for the Study of Diabetes (EASD) Diabetes Care 2012 35 1364 1379 10.2337/dc12-0413 22517736 PMC3357214

[B14] Tong PC Ko GT So WY Chiang SC Yang X Kong AP Ozaki R Ma RC Cockram CS Chow CC Chan JC Use of anti-diabetic drugs and glycaemic control in type 2 diabetes–The Hong Kong Diabetes Registry Diabetes Res Clin Pract 2008 82 346 352 10.1016/j.diabres.2008.09.006 18926583

[B15] Chan JC Gagliardino JJ Baik SH Chantelot JM Ferrera SR Hancu N Ilkova H Ramchandran A Aschner P Multifaceted determinants for achieving glycemic control: The international diabetes management practice study (IDMPS) Diabetes Care 2009 32 227 233 19033410 10.2337/dc08-0435 PMC2628684

[B16] Gagliardino JJ Aschner P Baik SH Chan J Chantelot JM Ilkova H Ramachandran A Patients' education, and its impact on care outcomes, resource consumption and working conditions: data from the International Diabetes Management Practices Study (IDMPS) Diabetes Metab 2012 38 128 134 10.1016/j.diabet.2011.09.002 22019715

[B17] US National Institute of Diabetes and Digestive and Kidney Diseases US National Institutes of Health NIH publication no: Diabetes in America, 2nd edition 95 1468 [http://diabetes.niddk.nih.gov/dm/pubs/america/contents.aspx]

[B18] Bi Y Zhu D Cheng J Zhu Y Xu N Cui S Li W Cheng X Wang F Hu Y Shen S Weng J The status of glycemic control: a cross-sectional study of outpatients with type 2 diabetes mellitus across primary, secondary, and tertiary hospitals in the Jiangsu province of China Clin Ther 2010 32 973 983 10.1016/j.clinthera.2010.05.002 20685506

