# Supplementary material for: Glycemic control among patients in China with type 2 diabetes mellitus receiving oral drugs or injectables
Source: BMC Public Health. 2013 Jun 21;13:602. doi: 10.1186/1471-2458-13-602 (PMC3729491; doi:10.1186/1471-2458-13-602)
Supplement: Additional file 1 — HbA1c Surveillance Registration Form. [file 1471-2458-13-602-S1.docx]

**Appendix.** HbA1c Surveillance Registration Form

**Instructions**

This questionnaire consists of 3 pages. The objective is to collect your general information, diabetes treatment information, and other relevant disease information. Please answer the following questions that are relevant to your diabetes status. Some questions might not be applicable to you, or some questions may seem similar, but please make sure to answer each question.

Please read each question and provide the answer that you believe is correct. If you are not sure how to respond, please give us your best answer in your opinion. All the information you provided will be confidential and will be used for scientific research. There will be no link between your information and your identity.

Collecting this information will not affect your current or future treatment.

This questionnaire will take you 10 minutes. Thank you for your participation and support.

**Participant signature:**

Has the subject met the following inclusion/exclusion criteria? Yes□_1_ No□_2_If “yes,” please go on with the survey. If “no,” please stop.

**Inclusion criteria**

1. Type 2 diabetes, male or female, age ≥18 years
2. Currently under one of the following treatments for diabetes: OAD only, OAD combined with insulin, or OAD combined with GLP-1 receptor agonists
3. At least one outpatient medical record for diabetes
4. Live in local area for at least 6 consecutive months
5. For community hospitals, a patient must have HbA1c examination from the same community hospital where he/she is recruited, and did not visit referral hospital in the last 3 months
6. The first 7 patients who visit the investigator each day will be eligible

**Exclusion criteria**

1. Secondary diabetes
2. Lifestyle intervention only
3. Chinese herbal medicine only
4. Insulin only
5. Inpatients
6. Type 1 diabetes
7. Pregnant, breast-feeding women
8. Mental incapacity or other reasons precluding adequate understanding or cooperation in the study

**Visit date (compulsory):** |___|____|____|___|year|___|___|month|___|___|day

**Basic information (compulsory):**

Sex: □_1_Male □_2_Female Age: |______| years Height: |______| cm Weight: |______| kg
SBP/DBP: |______|/|______|mmHg (still)
Lipid profile (latest in recent 3 months): TG: |___|___|.|___|___| mmol/L TC: |___|___|.|___|___| mmol/L
LDL: |___|___|.|___|___| mmol/L

**Date of diabetes diagnosis (compulsory):** |__|__|__|__|year|__|__|month

**Glycemic control at diagnosis (compulsory):**

HbA1c: |___|___|.|___|___|% FPG: |___|___|.|___|___| mmol/L 2hPPG: |___|___|.|___|___| mmol/L

**Current glycemic control (compulsory):**

| **Parameters** | **Values** | **Date** (latest in recent 3 months) |
| --- | --- | --- |
| HbA1c | \|___\|___\|.\|___\|___\|% | \|___\|____\|____\|___\|year\|___\|___\|month\|___\|___\|day\| |
| FPG | \|___\|___\|.\|___\|___\| mmol/L | \|___\|____\|____\|___\|year\|___\|___\|month\|___\|___\|day\| |
| 2hPPG | \|___\|___\|.\|___\|___\| mmol/L | \|___\|____\|____\|___\|year\|___\|___\|month\|___\|___\|day\| |
| Did you test your blood sugar in the last week? Yes□_1_ No□_2_ If “yes,” please fill in how many times you tested last week: \| \| time(s). | | |

**Treatment regimen^a^ (compulsory):**

| **Your current treatment regimen** | □_1_1 OAD only □_2_2 OADs □_3_3 OADs □_4_ ≥4 OADs  □_5_OAD+insulin □_6_OAD＋GLP-1 | |
| --- | --- | --- |
| **Please tick the drug you are using now**  (Please multi-select if you are using several OADs)  **The date of beginning of the treatment?**  \|___\|____\|____\|___\|year\|___\|___\|month\| | □_1.1_Gliclazid □_1.2_Glimepiride □_1.3_Glibenclamide □_1.4_ Xiaokewan [combination pill of glibenclamide and Chinese herb] □_1.5_Glipizide □_1.6_Gliquidone □_1.7_Repaglinide □_1.8_Nateglinide □_1.9_Mitiglinide □_1.10_Metformin □_1.11_Rosiglitazone □_1.12_Pioglitazone □_1.13_Acarbose □_1.14_Voglibose □_1.15_ Miglitol □_1.16_ Sitagliptin □_1.17_ GLP-1 (non-OAD) □_1.18_others (please specify) \|_______________________\| | |
| **If you are receiving OAD+insulin treatment, please tick the type of the insulin you are using**  (Please multi-select if you are using several insulins)  **The date of insulin initiation**  \|___\|____\|____\|___\|year\|___\|___\|month\|  **The reason for insulin initiation**  □_1_OAD ineffective  □_2_Complication  □_3_Patient requests  □_4_Other reason | Short-acting human insulin | □_2.1_Novolin R □_2.2_Humulin R □_2.3_Ganshulin R □_2.4_SciLin R □_2.5_Wanbanglin R [biosimilar] |
|  | Intermediate-acting human insulin | □_2.6_Humulin N □_2.7_Novolin N □_2.8_Ganshulin N □_2.9_SciLin N □_2.10_Wanbanglin N [biosimilar] |
|  | Premixes, human insulin | □_2.11_Novolin 30R □_2.12_Novolin50R □_2.13_Humulin 70/30 □_2.14_Ganshulin 30R □_2.15_SciLin M30 □_2.16_Wanbanglin 30R [biosimilar] |
|  | Rapid-acting insulin analogue | □_2.17_NovoRapid □_2.18_Humalog |
|  | Long-acting insulin analogue | □_2.19_Levemir □_2.20_Lantus □_2.21_Basalin |
|  | Insulin analogue premixes | □_2.22_ NovoMix 30 □_2.23_Humalog 25 □_2.24_ Humalog 50 |
|  | Animal insulin | Please specify the brand name\|_____________________________\| |
|  | Other insulin | Please specify the brand name\|_____________________________\| |
|  | **Total dosage of insulin per day: \|**_____________\| (U/day) | |

C**oncomitant disease (compulsory):**

| **Do you have the following disease(s)?** □_1_Yes □_2_No □_3_Don’t know If “yes,” please tick the diagnosis (multi-selectable) and date. | |
| --- | --- |
| □_1_ Hypertension | Diagnosis date: \|___\|____\|____\|___\|year\|___\|___\|month or □_3_Don’t know |
| □_2_ Coronary heart disease (angina pectoris, MI, etc) | Diagnosis date: \|___\|____\|____\|___\|year\|___\|___\|month or □_3_Don’t know |
| □_3_ Dyslipidemia | Diagnosis date: \|___\|____\|____\|___\|year\|___\|___\|month or □_3_Don’t know |
| □_4_ Cerebrovascular disease: cerebral infarction (stroke, hemiplegia, etc) | Diagnosis date: \|___\|____\|____\|___\|year\|___\|___\|month or □_3_Don’t know |
| □_5_ Diabetic retinopathy | Diagnosis date: \|___\|____\|____\|___\|year\|___\|___\|month or □_3_Don’t know |
| □_6_ Diabetic neuropathy | Diagnosis date: \|___\|____\|____\|___\|year\|___\|___\|month or □_3_Don’t know |
| □_7_ Diabetic nephropathy | Diagnosis date: \|___\|____\|____\|___\|year\|___\|___\|month or □_3_Don’t know |
| □_8_ Diabetic foot (uncured/unhealed ulcer) | Diagnosis date: \|___\|____\|____\|___\|year\|___\|___\|month or □_3_Don’t know |
| □_9_ Other diagnosed disease(s), please specify: _______________ | Diagnosis date: \|___\|____\|____\|___\|year\|___\|___\|month or □_3_Don’t know |

[^a^Note: drug names have been supplied in English where possible.]

**Investigator signature:**
